# Spatial Differentiation of Heavy Metals/Metalloids, Microbial Risk Genes and Soil Microbiota in a Sulfur-Contaminated Landscape

**DOI:** 10.3390/microorganisms13092010

**Published:** 2025-08-28

**Authors:** Lina Li, Jiayin Zhao, Chang Liu, Yiyan Deng, Yunpeng Du, Yu Liu, Yuncheng Wu, Wenwei Wu, Xuejun Pan

**Affiliations:** 1 Faculty of Environmental Science and Engineering, Kunming University of Science and Technology, Kunming 650500, China; 2 Yunnan Academy of Ecological and Environmental Sciences, Kunming 650034, China; 3 Nanjing Institute of Environmental Sciences, Ministry of Ecology and Environment, Nanjing 210042, China

**Keywords:** sulfur smelting, heavy metal and metalloids contamination, soil microbial communities, metagenomics, spatial distribution

## Abstract

Legacy sulfur smelting has left behind complex contamination landscapes, yet the spatial structuring of microbial risks and adaptation strategies across soil profiles remains insufficiently understood. Microbial risk genes, including those conferring resistance to antibiotic resistance (ARGs), biocide and metal resistance (BRGs/MRGs), and virulence (VFGs), are increasingly recognized as co-selected under heavy metal stress, posing both ecological and public health concerns. In this study, we integrated geochemical analyses with metagenomic sequencing and functional annotation to jointly characterize the vertical (0–7 m) and horizontal (~2 km) distribution of heavy metals/metalloids, microbial communities, and functional risk genes at a historic smelting site in Zhenxiong, Yunnan. Heavy metals and metalloids such as arsenic (As), chromium (Cr), copper (Cu), and lead (Pb) showed clear accumulation with depth, while significantly lower concentrations were observed in both upstream and downstream locations, revealing persistent vertical and horizontal pollution gradients. Correspondingly, resistance and virulence genes were co-enriched at contaminated sites, suggesting potential co-selection under prolonged stress. LEfSe analysis revealed distinct ecological patterns: vertically, upper layers were dominated by nutrient-cycling and mildly stress-tolerant taxa, while deeper layers favored metal-resistant, oligotrophic, and potentially pathogenic microorganisms; horizontally, beneficial and diverse microbes characterized low-contamination zones, whereas heavily polluted areas were dominated by resistant and stress-adapted genera. These findings provide new insights into microbial resilience and ecological risk under long-term smelting stress.

## 1. Introduction

Soil contamination resulting from prolonged industrial activities poses a significant threat to terrestrial ecosystems due to the persistence and toxicity of associated pollutants [1,2]. One well-documented case is from the early 1970s in El Paso, Texas, USA, where long-term mining and smelting activities caused severe heavy metal/metalloids contamination. This resulted in school closures, community relocation, and widespread damage to surrounding soils and vegetation [3]. Such cases highlight the long-lasting ecological and health impacts of smelting operations worldwide. Among such activities, sulfur and non-ferrous metal smelting operations generate substantial quantities of residues enriched in toxic elements such as arsenic (As), cadmium (Cd), lead (Pb), zinc (Zn), and copper (Cu) [4]. These residues are frequently deposited in open environments without effective containment, gradually leaching into the surrounding soils and creating complex contamination gradients both laterally and with depth [5,6]. These elements are highly toxic even at low concentrations and can impair soil fertility, inhibit plant growth, and cause oxidative stress in microorganisms. Beyond local soil contamination, their persistence and chemical stability allow them to enter food chains, where they may accumulate in organisms (bioconcentration) and amplify through trophic levels (biomagnification) [7]. Such processes not only prolong their environmental presence but also extend their risks to wildlife and human health [8,9]. In addition to these metals and metalloids, smelting processes also release non-metallic contaminants such as sulfur dioxide (SO_2_) and cyanide compounds [10]. These pollutants can exacerbate soil acidification, alter redox conditions, and increase ecological toxicity, thereby intensifying the overall environmental impact of smelting residues [11]. Moreover, sulfur biotransformation plays a critical role in shaping soil chemistry under smelting stress. Microbial oxidation of sulfide minerals generates sulfuric acid, which not only drives soil acidification but also promotes the leaching and mobilization of metals bound to sulfide phases [12]. These processes accelerate contaminant dispersion and amplify ecological risks by coupling sulfur cycling with heavy metal release.

Soil microorganisms are fundamental to ecosystem functioning, playing critical roles in nutrient cycling, organic matter decomposition, and pollutant detoxification [13]. However, microbial communities are highly sensitive to environmental stressors such as heavy metals/metalloids [14]. Numerous studies have demonstrated that metal/metalloid pollution can reduce microbial diversity, shift community composition toward stress-tolerant taxa, and disrupt microbial network connectivity, thereby weakening ecosystem resilience [14,15]. On the other hand, certain microbial lineages possess specific adaptations that allow them to persist under metal/metalloid stress, including metal efflux mechanisms, antioxidant defenses, and horizontal gene transfer capabilities [16]. Such adaptations lead to selective enrichment of resilient taxa and functional traits under long-term contamination. An emerging concern is that chronic heavy metal/metalloid exposure may co-select for genes related to antibiotic resistance (ARGs), biocide and metal resistance (BRGs/MRGs), and virulence (VFGs) [17]. Heavy metal/metalloid contamination exerts selective pressure that favors microorganisms carrying detoxification systems such as efflux pumps, metal-binding proteins, and enzymatic reduction pathways [18]. These mechanisms not only enhance metal tolerance but are frequently co-localized with antibiotic resistance and virulence determinants on mobile genetic elements. As a result, cross-resistance and co-selection occur, whereby exposure to metals indirectly enriches antibiotic resistance genes and virulence factors. This phenomenon enhances the risk of disseminating antimicrobial resistance and opportunistic pathogens from polluted environments into broader ecological and public health contexts [19]. Elevated levels of ARGs and VFGs have been reported in soils near smelting and mining sites, highlighting their potential role as reservoirs of resistance and virulence traits [20,21]. Monitoring microbial risk genes in contaminated soils is particularly critical in the framework of One Health. Soil functions as both a sink and a source of resistance and virulence determinants, and the dissemination of these genes across ecological compartments increases the risk of transfer to pathogens that affect agriculture, livestock, and human populations. Evaluating their spatial dynamics under long-term contamination is therefore essential for anticipating biosafety risks and guiding sustainable management strategies. Despite these insights, most previous studies have focused on surface soils or single transects, often neglecting the spatial heterogeneity introduced by both vertical stratification and horizontal dispersion of contaminants [22,23]. In reality, long-term residue deposition often results in steep gradients not only with depth but also across lateral distances from pollution sources, potentially leading to divergent microbial responses across different spatial scales. A comprehensive analysis of both vertical and horizontal patterns is therefore necessary to fully understand the ecological dynamics and biosafety implications of heavily polluted sites.

In this study, we investigated a sulfur smelting waste disposal site in Zhenxiong County, Yunnan Province, where smelting activities have persisted for over four decades. To capture the full extent of spatial heterogeneity, we conducted a dual-gradient sampling approach that included both vertical (depth profile from 0–7 m) and horizontal (spatial transect up to 1.5 km from the residue pile) directions. Using metagenomic sequencing combined with functional gene annotation and geochemical profiling, this study aimed to: (1) evaluate the spatial distribution of heavy metals/metalloids and associated changes in soil physicochemical properties along vertical and horizontal gradients surrounding a historical sulfur smelting site; (2) investigate the abundance and distribution patterns of resistance genes (ARGs, BRGs, MRGs) and virulence genes (VFGs), and examine their co-occurrence with pollution intensity; and (3) characterize shifts in microbial community structure, diversity, and biomarker taxa, and explore their ecological strategies under varying contamination pressures. By integrating both vertical and horizontal dimensions of environmental stress, this study offers a comprehensive view of microbial adaptation, ecological risk, and potential targets for bioremediation in complex contaminated soils.

## 2. Materials and Methods

### 2.1. Soil Sample Collection

Soil samples were collected from a long-term sulfur smelting waste residue disposal site in Zhenxiong County, Yunnan Province, China (105°12′ E, 27°30′ N). The site has experienced continuous accumulation of sulfur-rich smelting slag for nearly four decades and spans approximately 12,500 m^2^ (Figure S1). It harbors over 410,000 tons of waste residues, primarily contaminated with As, Cr, Cd, Cu, Ni, and Pb. To investigate the vertical and horizontal variation in soil properties, microbial communities, and contaminant profiles, a stratified sampling strategy was adopted. Before sampling, surface vegetation, litter, and visible debris were carefully removed to avoid external contamination. At the smelting deposit zone, deposited residues were thoroughly cleared away, after which soils were collected using stainless-steel augers, with gloves changed between samples to prevent cross-contamination. For vertical gradient analysis, samples were collected from the waste deposit at four soil depth intervals: 0–0.5 m (D1), 0.5–2 m (D2), 2–4 m (D3), and 4–7 m (D4). At each depth, three replicate composite samples were obtained, each generated by thoroughly mixing subsamples from five nearby drilling points to capture spatial heterogeneity, resulting in a total of 12 vertical samples. For horizontal spatial analysis, soil samples were collected from four locations along a potential flow path influenced by smelting runoff: an upstream control site located 500 m from the deposit (L1), the immediate smelting deposit zone (L2), a downstream site 500 m from the deposit, and a more distal site approximately 1.5 km downstream (L4). At each location, soil was sampled from 0–1 m depth, mixed to form a composite sample, and replicated across three sites, yielding an additional 12 horizontal samples. At each location, 0–1 m soil cores were collected in triplicate using the five-point method, composited into one representative sample per replicate, and processed as above, yielding an additional 12 horizontal samples.

### 2.2. Soil Physicochemical Analysis

Soil pH was measured in a 1:2.5 (*w*/*v*) soil/water suspension using a pH meter (PHS-3C, INESA, Shanghai, China). Organic matter (OM) was determined using the potassium dichromate oxidation method. Total nitrogen (TN) was analyzed by the Kjeldahl digestion method. Available nitrogen (AN) was measured using the alkaline hydrolysis diffusion method. Total phosphorus (TP) was determined by molybdenum blue colorimetry after HClO_4_-H_2_SO_4_ digestion, and available phosphorus (AP) was extracted using the Olsen method and quantified by spectrophotometry. Total potassium (TK) was measured after acid digestion using a flame photometer (FP640, INESA, Shanghai, China).

Total concentrations of different heavy metals/metalloids were quantified to assess contamination levels. For each sample, 0.5 g of air-dried and sieved soil was digested using a tri-acid mixture consisting of concentrated nitric acid (HNO_3_), hydrochloric acid (HCl), and hydrofluoric acid (HF) in a volumetric ratio of 5:3:2. The digestion was carried out in a closed-vessel microwave digestion system (Mars 6, CEM Corporation, Matthews, NC, USA) to ensure complete mineral dissolution under controlled pressure and temperature conditions. After digestion, the solutions were cooled, filtered through a 0.22 µm membrane, and diluted to a final volume of 50 mL with ultrapure water. The concentrations of As, Cr, Cd, Cu, Ni, and Pb were then determined using inductively coupled plasma mass spectrometry (ICP-MS) (Thermo, Waltham, MA, USA) following quality assurance procedures with calibration standards and procedural blanks [24]. To ensure analytical accuracy, certified reference material (GBW07405, Institute of Geophysical and Geochemical Exploration, Beijing, China) was analyzed in parallel with the soil samples, and recovery rates for all measured elements were within acceptable ranges (90–110%). All analyses were performed in triplicate to ensure accuracy and reproducibility.

### 2.3. Metagenome Sequencing and Analysis

DNA was extracted from a total of 24 composite soil samples (12 vertical and 12 horizontal) using the DNeasy PowerSoil Kit (Qiagen, Hilden, Germany) according to the manufacturer’s instructions. To ensure the quality of downstream sequencing, DNA concentration and purity were assessed using a NanoDrop 2000 spectrophotometer (Thermo, Waltham, MA, USA). Metagenomic libraries were prepared using standard Illumina protocols. Paired-end sequencing (150 bp × 2) was performed on the Illumina NovaSeq 6000 platform (Illumina, San Diego, CA, USA), generating an average of 20 Gb of raw sequencing data per sample to ensure sufficient coverage of soil microbial communities. The raw reads obtained in this study were submitted to the National Center for Biotechnology Information (NCBI) under the accession number PRJNA1307676. Raw reads were subjected to quality control using Fastp (v0.23.2) [25], which performed adapter trimming, low-quality base removal (Q-score < 20), and filtering of reads shorter than 50 bp. Clean reads passing quality control were used for both taxonomic and functional profiling. For taxonomic classification, Kraken2 (v2.1.2) was employed with a custom database built from the NCBI RefSeq genome collection. This database integrates high-quality, non-redundant reference sequences curated by NCBI, thereby providing broad taxonomic coverage across major microbial domains. Taxonomic identities of metagenomic reads were assigned at multiple levels based on exact k-mer matches to this database. Bracken (v2.9) was then applied to re-estimate species-level relative abundances by refining Kraken2 outputs using the read length–specific k-mer distribution of the RefSeq sequences.

Metagenomic reads were assembled de novo using MEGAHIT (v1.2.9) with the “meta-large” preset, corresponding to parameters --k-min 27 --k-max 127 --k-step 10. Contigs shorter than 500 bp were discarded to reduce spurious assemblies. Assembly quality was assessed based on summary statistics provided by MEGAHIT, including N50, total assembly length, and maximum contig size, which were used as criteria to confirm the reliability of the final assemblies used in downstream analyses [26]. Open reading frames (ORFs) were predicted from assembled contigs using Prodigal (v2.6.3) in metagenomic mode [27]. Redundant protein-coding sequences were clustered using CD-HIT (v4.8.1) at 95% sequence identity to generate a non-redundant unigene catalog for downstream analysis [28]. Gene abundance quantification was performed using Salmon (v1.10.1). Clean reads from each sample were mapped to the non-redundant unigene set, and abundances were expressed as TPM (transcripts per million) values, calculated by normalizing read counts to both gene length and the total number of mapped reads per sample. Here, TPM refers to normalized DNA-based gene abundance values. Functional annotation of resistance and virulence genes was performed using BLASTP (v2.13.0) with an E-value cutoff of 1 × 10^−10^. Antibiotic resistance genes (ARGs) were identified by aligning unigenes to the Comprehensive Antibiotic Resistance Database (CARD) [29]. Biocide and metal resistance genes (BRGs and MRGs) were annotated using the BacMet2 experimentally confirmed resistance gene database [30]. Virulence factor genes (VFGs) were annotated against the Virulence Factor Database (VFDB) [31].

### 2.4. Statistical Analysis

Statistical analyses were conducted using IBM SPSS Statistics (v26.0). One-way analysis of variance (ANOVA) was used to evaluate differences in horizontal and vertical distributions with significance set at *p* < 0.05. For post hoc pairwise comparisons, Tukey’s HSD test was applied, which corrects for multiple testing. Unless otherwise specified, data visualization was performed in R (v4.4.1). Alpha diversity indices including Shannon and Chao1, were calculated using the vegan packages. Beta diversity was assessed using principal coordinate analysis (PCoA) based on Bray–Curtis dissimilarity matrices, followed by analysis of similarities (ANOSIM) to test for significant differences in microbial community composition across groups [32]. To identify taxonomic biomarkers associated with different depths and horizontal sites, linear discriminant analysis effect size (LEfSe) was performed using the OmicStudio tools at https://www.omicstudio.cn/tool (accessed on 20 May 2025). Redundancy analysis (RDA) was conducted using the vegan package in R (v4.3.1) to evaluate the relationships between microbial community composition and soil physicochemical variables.

## 3. Results and Discussion

### 3.1. Distribution of Heavy Metals/Metalloids and Soil Properties

According to previous investigations, As, Cr, Cd, Cu, Ni, and Pb were identified as the primary pollutants based on their concentrations exceeding local background values and risk screening thresholds. The vertical distribution of heavy metal and metalloid content at the long-term sulfur smelting waste residue disposal site was shown in Table 1. A general increasing trend in As, Cr, Cd, and Ni concentrations with soil depth was observed across the profile. Surface layers (D1 and D2) exhibited comparatively lower concentrations than subsurface layers (D3 and D4). These findings are consistent with earlier studies conducted in industrial zones of northeastern China. For example, Huang et al. [33] observed a substantial accumulation of Cr at 40–60 cm depth, exceeding the levels detected at shallower layers (0–20 cm). This vertical enrichment trend indicates a pronounced leaching effect, likely driven by long-term infiltration of metal-rich percolates under acidic and oxidizing conditions associated with sulfur smelting, which enhance heavy metal/metalloid solubility and downward transport [5]. Moreover, individual metal/metalloid exhibited distinct vertical distribution patterns, reflecting differences in their chemical properties, mobility, and interactions with soil components. For instance, As, Cd, Cr, and Ni exhibited substantially greater enrichment in subsurface layers (D3 and D4) compared to Cu and Pb, indicating their higher vertical mobility. Cd and Ni are known for their relatively high solubility in low-pH environments, where they remain in ionic forms and are less likely to precipitate or strongly bind to soil colloids, thereby facilitating their leaching into deeper layers [34,35]. In contrast, Cu and Pb tend to form stable complexes with organic matter and are more strongly adsorbed onto mineral surfaces, which limits their mobility and results in their preferential retention in surface soils [36,37].

The vertical distribution of heavy metals/metalloids was also linked to key soil physicochemical properties [5,38]. The entire soil profile exhibited strongly acidic conditions, with pH values ranging from 4.03 to 5.02 (Table 2). Such persistent acidity is likely a result of long-term sulfur smelting activities, which have led to chronic acidification. Acidic environments accelerate the weathering of primary minerals and enhance the desorption of heavy metals/metalloids from soil colloids and oxides, thereby increasing their solubility and promoting downward migration [39]. This mechanism likely contributes to the observed enrichment of metals/metalloids in deeper soil layers. In addition, while organic matter (OM), total nitrogen (TN), and total phosphorus (TP) remained relatively stable with depth, notable variations were observed in the distribution of available nutrients. Available phosphorus (AP) increased gradually with depth, whereas available nitrogen (AN) decreased. The downward trend of AN likely reflects its association with microbial mineralization processes concentrated in surface layers and the susceptibility of nitrogenous compounds to leaching losses. In contrast, the enrichment of AP in deeper layers may be due to its weaker adsorption in highly acidic soils and its migration along with infiltrating water [40]. Overall, the vertical stratification of heavy metals/metalloids reflects a complex interplay among metal-specific geochemical behaviors, soil physicochemical properties, and site-specific environmental conditions.

To further elucidate spatial contamination patterns, the horizontal distribution of heavy metals/metalloids and soil properties was analyzed across four representative sites (Table 1). The results revealed distinct spatial heterogeneity in both pollutant accumulation and soil characteristics. As expected, the upstream site (L1) exhibited the lowest concentrations of all measured metals/metalloids. In contrast, metal/metalloid concentrations at the smelting deposit zone (L2) were significantly elevated, reflecting direct contamination from long-term deposition and surface runoff of metal-rich particulates. Notably, the Cd concentration was exceptionally high at the first downstream site (L3), suggesting that Cd is highly mobile and prone to transport through surface runoff or subsurface flow [41]. Ni also remained elevated at both L3 and L4, indicating sustained downstream mobility and potential for longer-range dispersion. Although the concentrations of Cu, Cr, and Pb at L3 and L4 were slightly lower than those at L2, their continued presence downstream underscores the persistence of these metals in the surrounding landscape. The observed attenuation may be attributed to dilution, sediment deposition, or partial immobilization by soil components [42,43]. Overall, the metal/metalloid concentrations at L3 and L4 exceeded those at L1 by factors ranging from 1.04 to 18.15. These findings underscore the spatial extent of contamination, demonstrating that smelting-related discharges influence not only the immediate vicinity of the source but also downstream areas up to 1.5 km away.

The horizontal variation in pH strongly reflected the influence of sulfur smelting contamination and played a critical role in shaping metal/metalloid mobility and soil chemistry. The lowest pH value was observed at the smelting deposit zone (Table 2), indicating intense acidification caused by long-term exposure to sulfur-derived compounds. In contrast, higher pH values were recorded at downstream sites L3 and L4, which may reflect natural buffering capacity from riverine sediment inputs or decreased acid deposition with increasing distance from the pollution source. These more neutral conditions could facilitate partial metal/metalloid immobilization through precipitation or increased adsorption, contributing to the observed attenuation of some metal/metalloid concentrations downstream [44]. Organic matter remained relatively stable across sites, indicating limited disturbance to carbon inputs or decomposition processes. In contrast, total nitrogen (TN), available nitrogen (AN), and available phosphorus (AP) exhibited spatial variability, likely influenced by differences in pH, microbial activity, and hydrological transport. Higher nutrient levels at L1 and L3 may reflect more favorable pH conditions that support microbial mineralization and nutrient retention, while their lower values at L2 may result from acid-induced microbial inhibition and nutrient leaching under strong acid stress [45,46]. This highlights the importance of soil acidity in driving metal mobility, leading to vertical enrichment of As, Cr, Cd, and Ni and downstream transport of Cd and Ni, which together pose long-term ecological risks in sulfur-smelting soils.

### 3.2. Profiles of Resistance and Virulence Genes

**Figure 1:**
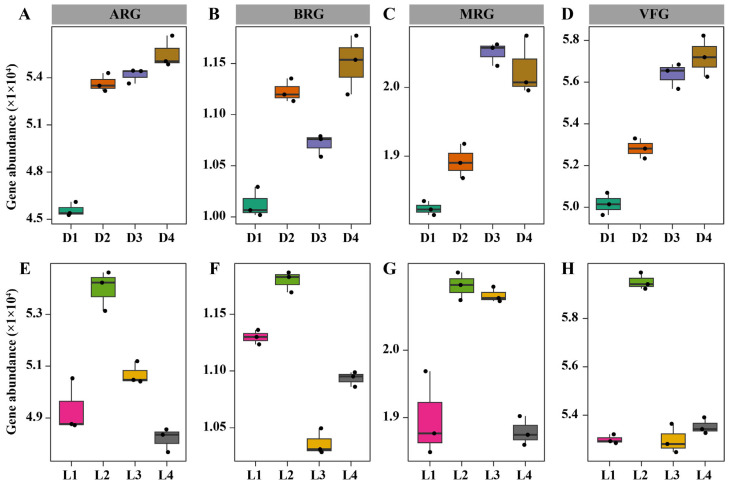
Abundance of resistance genes (ARGs, BRGs and MRGs) and virulence genes (VFGs). Panels (**A**–**D**) show vertical distributions across soil depths: 0–0.5 m (D1), 0.5–2 m (D2), 2–4 m (D3), and 4–7 m (D4). Panels (**E**–**H**) show horizontal distributions across four sites: L1 (an upstream control site, 500 m from the smelting deposit), L2 (the immediate smelting deposit zone), L3 (a downstream site, 500 m from the deposit), and L4 (a distal downstream site, approximately 1.5 km from the deposit).

Long-term metal/metalloid pollution poses significant threats to soil ecosystems, including the enrichment of microbial resistance and virulence traits [47]. To evaluate these risks, we analyzed the distribution of ARGs, BRGs, MRGs, and VFGs across both vertical and horizontal gradients (Figure 1). Along the vertical profile, the relative abundance of all four gene categories increased with depth (Figure 1A–D), which coincided with elevated concentrations of most heavy metals/metalloids. These deeper layers, which are characterized by higher metal/metalloid loads and persistent acidity, likely exert strong selective pressure that favors microbial taxa harboring metal/metalloid tolerance and co-resistance traits. The concurrent enrichment of ARGs and MRGs supports the concept of co-selection, where metal/metalloid stress indirectly promotes antibiotic resistance through genetic linkage or shared regulatory pathways [48,49]. Similarly, the increased abundance of VFGs in deeper soils may reflect both shifts in microbial community composition and the activation of virulence mechanisms under chemical stress, potentially elevating the ecological risk posed by opportunistic soil pathogens [50]. Across the horizontal transect, the highest gene abundances were observed at the smelting deposit zone (L2) (Figure 1E–H), where metal/metalloid concentrations and soil acidity were most severe. This enrichment is likely a direct response to the intense environmental pressure imposed by long-term smelting residue exposure, which may favor microbial taxa capable of resisting toxic compounds and expressing virulence-related traits for survival under stress [51]. Although gene abundances declined at downstream sites (L3 and L4), ARGs, MRGs, and VFGs remained elevated compared to the upstream control site (L1). The sustained presence of resistance and virulence genes at distal sites implies that hydrological transport and microbial dispersal may contribute to the spread of risk genes throughout the broader landscape.

**Figure 2:**
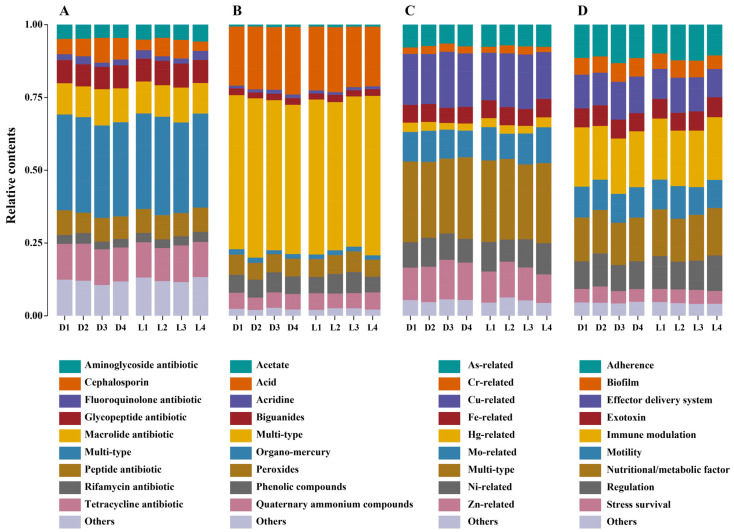
Composition and distribution of resistance and virulence gene subtypes. Panels (**A**–**C**) display the relative abundance of subtypes for ARGs, BRGs, and MRGs, respectively. Panel (**D**) shows the subtype distribution of VFGs.

To gain a deeper insight into functional adaptation, we further examined the dominant subtypes within each gene category (Figure 2). Among ARGs, multidrug resistance genes were consistently the most abundant across all samples (Figure 2A, Figures S2 and S3), which underscored the widespread presence of broad-spectrum resistance mechanisms. Genera such as *Pseudomonas*, *Burkholderia*, *Cupriavidus*, *Streptomyces*, and *Mycobacterium* are well known for their capacity to harbor diverse multidrug efflux systems. These are primarily mediated by efflux pumps, which actively expel a wide range of toxic compounds from the cell, and global regulators, which coordinate stress responses and activate multiple resistance pathways simultaneously [52]. Their pronounced enrichment in deeper soil layers (D3–D4) and at the smelting deposit zone (L2) supports the hypothesis that long-term heavy metal/metalloid stress exerts co-selection pressure favoring the persistence of multidrug-resistant microorganisms. In addition to multi-type ARGs, resistance genes against tetracyclines, macrolides, and glycopeptides were frequently detected, with elevated abundance in both deeper layers and downstream sites (L3–L4). These classes of ARGs act through diverse mechanisms. Tetracycline resistance is mainly achieved by efflux pumps that remove the drug from the cell or by proteins that protect the ribosome; macrolide resistance often involves methyltransferases that modify ribosomal RNA and block drug binding; and glycopeptide resistance usually occurs through enzymes that alter the cell wall target, reducing drug affinity. These gene subtypes are commonly associated with mobile genetic elements and may persist in the soil environment even in the absence of direct antibiotic inputs [53]. The marked accumulation of cephalosporin resistance genes at D3–D4 and L2 further suggests localized enrichment of β-lactamase-producing bacteria which inactivate β-lactam antibiotics by hydrolyzing the β-lactam ring, in response to contamination stress. Their decline at L4 may reflect downstream dilution or reduced selection pressure [54]. In contrast, fluoroquinolone and rifamycin resistance genes displayed more variable spatial patterns. Fluoroquinolone resistance is commonly mediated by mutations in DNA gyrase and topoisomerase IV or by efflux pumps that reduce intracellular drug levels, while rifamycin resistance typically arises from mutations in the *rpoB* gene encoding the RNA polymerase β-subunit, which prevent drug binding. Their relatively high abundance at the middle layer (D2) and at peripheral locations (L1, L4) may reflect influences unrelated to metal/metalloid contamination, such as legacy agricultural antibiotic use or intrinsic resistance among indigenous microbial taxa. Within BRGs (Figure 2B, Figures S4 and S5), multi-type resistance genes consistently dominated, indicating the widespread presence of metabolic and transport systems conferring resistance to multiple biocidal agents. These were particularly enriched in deeper layers (D4) and highly abundant at L2, consistent with adaptive survival in chemically stressed environments. Subtypes such as acid, peroxide, and phenolic compound resistance genes were highly represented, especially in acidic soils near the smelting residue. These genes were mainly associated with genera such as *Acidithiobacillus* and *Leptospirillum*, which are well adapted to acid mine environments, as well as *Pseudomonas* and *Bacillus*, which are known for their oxidative stress response and detoxification capacities [55]. Their abundance suggests microbial reliance on stress defense pathways to cope with low pH and oxidative conditions [56]. Interestingly, resistance genes to quaternary ammonium compounds (QACs) and organo-mercury were observed high at the upstream control, indicating possible legacy pollution or horizontal spread across broader spatial scales. MRG subtype patterns strongly reflected site-specific metal/metalloid profiles (Figure 2C, Figures S6 and S7). Among all subtypes, multi-type MRGs were consistently the most abundant with peak levels observed in both deeper soil layers (D3–D4) and at the smelting deposit zone (L2). This suggests that microorganisms in heavily contaminated environments are likely to harbor operons or gene clusters conferring resistance to multiple metals/metalloids simultaneously so as to enable survival under complex metal/metalloid stress. Cu- and Cr-related resistance genes also exhibited high abundance, especially in subsurface soils (D3–D4) and at L2. This alignment strongly supports the role of these metals/metalloids as primary selective agents shaping MRG profiles in smelting-impacted soils. Although resistance genes specific to Cd and Pb were not among the most dominant subtypes, their resistance is often functionally linked to Zn-related resistance mechanisms due to co-regulation and shared transport systems (e.g., Czc/Cad operons) [57,58]. Such operons are well described in genera including *Pseudomonas*, *Ralstonia*, and *Cupriavidus*, which use Czc efflux systems to extrude Zn, Cd, and Co, as well as in *Bacillus* and other Firmicutes that harbor Cad operons conferring Pb and Cd resistance. The observed co-enrichment of Zn-related genes indirectly supports the persistence of Cd and Pb resistance capacity within the microbial community. In contrast, resistance genes associated with Hg, Mo, and Fe were less strongly correlated with measured metal levels and exhibited more uniform distributions across depths and sites. This may be due to their lower overall abundance in the contaminated matrix or their reduced ecological relevance in this specific pollution context. Virulence-related genes were diverse and abundant, particularly those associated with immune modulation, nutritional/metabolic function, and motility (Figure 2D, Figures S8 and S9). These subtypes were consistently dominant across both depth and distance gradients, suggesting that microbial communities have developed robust adaptive mechanisms to persist in metal/metalloid-stressed, acidic soils. Genes encoding effector delivery systems, biofilm formation, and adherence showed notable enrichment in subsurface layers (D3–D4) and at L2, indicating that contaminated zones may foster the selection of microbes with increased pathogenic potential or survival traits linked to environmental stress adaptation [59]. The elevated presence of biofilm and stress survival genes at L2 and L3 also implies enhanced microbial aggregation and resilience under chemical stress [60].

To explore the relationship between environmental contamination and microbial functional potential, we performed a Spearman correlation analysis between metal concentrations and resistance/virulence gene subtypes (Figure S10). Several statistically significant correlations (*p* < 0.05) were observed. Among them, As, Cr, and Cd showed the strongest positive associations as they exhibited strong correlations with multiple ARG subtypes, including multidrug, glycopeptide, cephalosporin, and macrolide resistance, as well as Zn-related MRGs. Cr also displayed broad and significant positive correlations, particularly with multidrug, macrolide, and β-lactam resistance genes, suggesting that Cr contamination acts as a major selective agent. Cd further correlated with ARG subtypes such as tetracycline and glycopeptide resistance, reflecting its potential role in promoting cross-resistance. These results indicate that As, Cr, and Cd are key selective pressures in sulfur-smelting soils, strongly influencing the abundance and distribution of resistance and virulence gene subtypes.

### 3.3. Patterns of Microbial Community Structure and Composition

**Figure 3:**
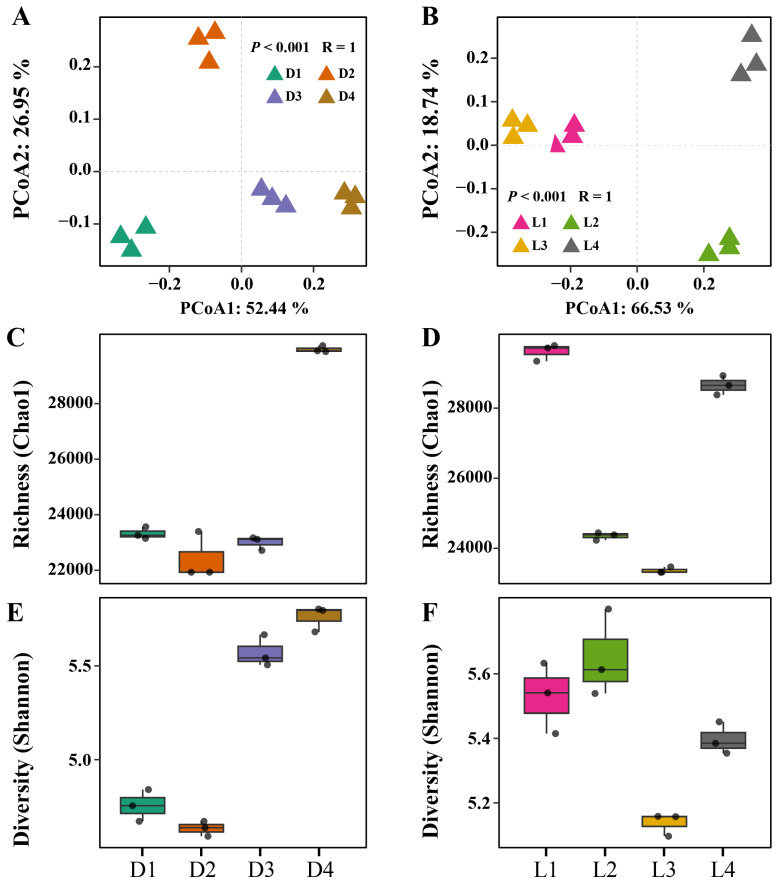
Microbial community diversity across vertical and horizontal gradients. (**A**,**B**) Principal coordinate analysis (PCoA) based on Bray–Curtis dissimilarity, showing community composition patterns along the vertical (**A**) and horizontal (**B**) profiles. (**C**,**D**) Microbial richness estimated by Chao1 index for vertical (**C**) and horizontal (**D**) distributions. (**E**,**F**) Microbial diversity assessed by Shannon index for vertical (**E**) and horizontal (**F**) distributions.

Principal coordinate analysis (PCoA) revealed clear spatial segregation of microbial communities across both vertical and horizontal gradients (Figure 3A,B). Distinct clustering patterns were observed for each group, indicating significant differences in overall community composition. This was supported by ANOSIM results (R = 1, *p* < 0.001), which further suggested that metal/metalloid contamination and spatial separation exert a strong influence on microbial community structure. Along the vertical profile (D1–D4), microbial richness (Chao1) was lower in surface and mid-depth layers (D1–D3) and peaked in the deepest layer (D4) (Figure 3C). Shannon diversity followed a similar trend, with notably lower values in surface soils (D1–D2) and increasing diversity at greater depths (D3–D4) (Figure 3E). This might be because low to moderate levels of heavy metal/metalloid stress can enhance microbial diversity, possibly by suppressing dominant taxa and allowing less competitive species to coexist. In this study, while heavy metals/metalloids were present, the pollution levels in deeper layers may not have been severe enough to inhibit microbial growth outright but rather fostered a functionally diverse and stress-adapted community. Horizontally, Shannon diversity was highest at the smelting deposit zone (L2), which aligns with the hypothesis that intermediate levels of stress can increase evenness and niche differentiation among microbial taxa. In contrast, microbial richness was greater at the peripheral sites (L1 and L4). This discrepancy may be attributed to the broader spatial scale of the horizontal gradient (~2 km) compared to the vertical profile (~7 m). At this scale, additional environmental factors such as soil moisture, vegetation cover, land use practices, and hydrological connectivity likely play a significant role in shaping microbial richness and overall community composition.

**Figure 4:**
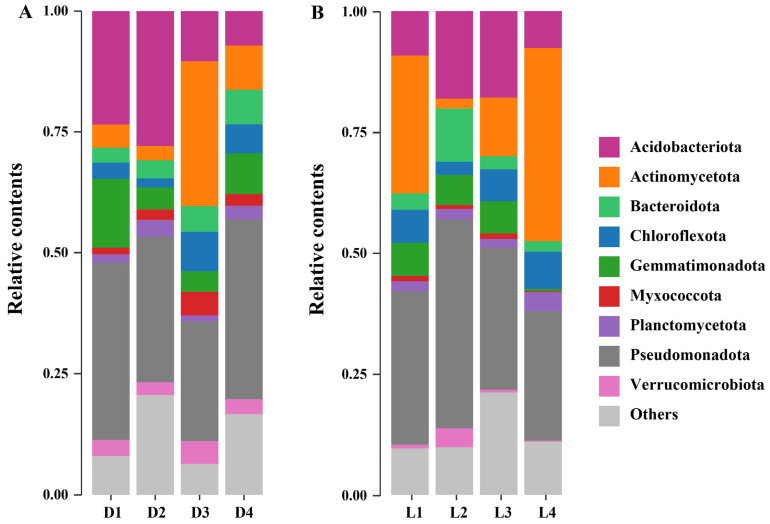
Phylum-level composition of soil microbial communities. Relative abundance of dominant phyla along the vertical layers (**A**) and horizontal transects (**B**).

Phylum-level microbial community profiles revealed distinct taxonomic shifts along both vertical and horizontal gradients. Across all samples, Pseudomonadota, Acidobacteriota, Actinomycetota, and Bacteroidota emerged as the most dominant phyla, with relative abundances ranging from 24.70–43.20%, 7.16–27.95%, 2.05–40.01%, and 2.17–11.02%, respectively (Figure 4). These findings are in line with previous studies. For instance, Zhao et al. [61] reported that Pseudomonadota (41.7%), Bacillota (20.4%), Acidobacteriota (9.3%), and Bacteroidota (8.2%) dominated microbial communities in mining areas with high heavy metal/metalloid concentrations. Similarly, Tipayno et al. [62] observed that Pseudomonadota (34.3%), Chloroflexota (19.0%), Acidobacteriota (14.6%), and Bacteroidota (10.5%) were the most abundant phyla in paddy soils contaminated by non-ferrous smelter activity. These parallels suggest that metal/metalloid-polluted environments across different geographical regions tend to select for similar microbial assemblages, highlighting the ecological filtering effect imposed by heavy metal/metalloid stress. Specifically, Pseudomonadota emerged as the most dominant phylum across all sites. This group is known for its high metabolic versatility, rapid growth rates, and tolerance to environmental stressors [63]. Members of Pseudomonadota often harbor metal resistance genes and efflux systems, enabling them to thrive in contaminated environments and potentially act as reservoirs or vectors for horizontal gene transfer of resistance traits [64]. Acidobacteriota represents another prevalent bacterial phylum in environmental systems. They are well adapted to acidic and oligotrophic conditions, which are characteristic of soils affected by sulfur smelting activities [65,66]. In this study, their relative abundance was higher in surface and moderately contaminated soils (D1 and D2), consistent with their ecological preferences. However, their abundance declined sharply in deeper layers (D3–D4), likely due to increased heavy metal/metalloid concentrations that exceed their tolerance thresholds. In contrast, Actinomycetota exhibited an opposite distribution pattern, with relatively low abundance in surface soils (D1–D2) and a sharp increase in deeper layers (D3–D4). This trend may be attributed to their well-documented capacity to withstand extreme environmental stressors, including heavy metal/metalloid toxicity and nutrient limitation [67]. Members of Actinomycetota are known for their robust cell wall structures, production of metal/metalloid-chelating compounds, and extensive secondary metabolism, all of which contribute to their resilience under high metal/metalloid load conditions [68]. Bacteroidota, Chloroflexota, and Myxococcota had relatively lower overall abundance but showed a notable increase in deeper soil layers (D3–D4), similar to the trend observed for Actinomycetota. This pattern suggests that these taxa may be better suited to the challenging conditions in subsurface soils. Bacteroidota are often involved in degrading complex organic compounds, which may still be present in deeper layers as recalcitrant matter [69]. Chloroflexota possess metabolic flexibility and can perform anaerobic respiration, which likely supports their survival in the low-oxygen, metal-rich environments of deep smelting-affected soils [70]. Myxococcota are known for their environmental resilience and ability to prey on other microbes, which may give them a competitive advantage under stress conditions [71]. Their presence in the deeper layers reflects ecological niche differentiation and the emergence of specialized microbial groups capable of maintaining functional roles despite long-term contamination.

**Figure 5:**
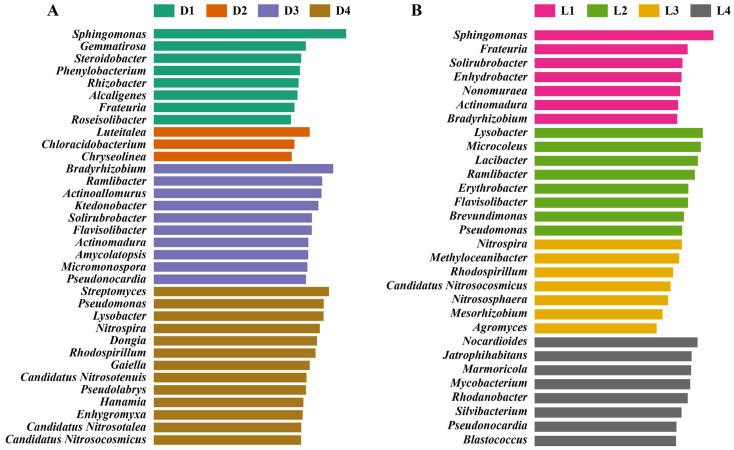
LEfSe analysis identifying differentially abundant microbial taxa. Biomarker taxa enriched across soil vertical layers (**A**) and horizontal transects (**B**).

LEfSe analysis revealed distinct biomarker taxa along both vertical and horizontal gradients. A clear compositional divergence was observed between the different depth layers (Figure 5A). In the upper layers (D1–D2), enriched taxa were primarily associated with copiotrophic or mildly stress-tolerant ecotypes. Genera such as *Sphingomonas*, *Gemmatirosa*, and *Steroidobacter* were significantly overrepresented. *Sphingomonas* is known for its metabolic flexibility and capacity to degrade aromatic pollutants, but also carries diverse resistance and virulence traits, posing a risk of harboring opportunistic pathogens in mildly polluted environments [72]. *Alcaligenes* and *Phenylobacterium* were also identified as biomarkers, with reported involvement in organic compound transformation and tolerance to oxidative stress [73]. The presence of these genera likely reflects the relatively moderate ecological pressure in D1–D2, where metal/metalloid concentrations have not yet reached inhibitory thresholds. In contrast, D3–D4 layers, subjected to prolonged and more severe contamination, showed enrichment of taxa associated with metal/metalloid resistance, nitrification, oligotrophy, and potentially pathogenic characteristics. Several Actinomycetota members were dominant in D3, including *Actinoallomurus*, *Actinomadura*, *Amycolatopsis*, *Micromonospora*, and *Pseudonocardia*. These are consistent with their roles in antibiotic production, spore formation, and resistance to environmental stress [74,75,76]. Their proliferation suggests a shift toward resilient taxa with biosynthetic and defensive advantages. In D4, taxa such as *Lysobacter*, *Rhodospirillum*, *Dongia*, and *Pseudomonas* were enriched. Those strains are known for their opportunistic pathogenicity, resistance traits, or biofilm formation abilities [77,78]. The co-occurrence of these genera with high metal/metalloid concentrations points to the selection of microbial populations that may not only tolerate but exploit stressful niches, posing potential ecological and public health concerns.

Horizontally (Figure 5B), in the low-contamination zones (L1 and L4), the microbiota was enriched with genera typically associated with nutrient cycling, stress mitigation, and plant-microbe interactions. Notably, *Sphingomonas*, *Frateuria*, *Solirubrobacter*, *Streptomyces*, *Actinomadura*, and *Bradyrhizobium* were significantly overrepresented in L1, while L4 showed increased abundance of *Nocardioides*, *Jatrophihabitans*, *Mycobacterium*, and *Pseudonocardia*. These taxa have been widely documented for their involvement in organic matter decomposition, nitrogen fixation, and biosynthesis of secondary metabolites. For instance, *Bradyrhizobium* and *Frateuria* play critical roles in rhizosphere nutrient dynamics [79], while *Streptomyces* and *Actinomadura* are known producers of antibiotics and antifungal compounds [75,80]. The presence of these genera in both upstream and distal downstream sites suggests that microbial communities in these zones retain functional traits typical of unpolluted or recovering soils, with relatively limited ecological disturbance. By contrast, the smelting deposit zone (L2) displayed enrichment of genera associated with stress tolerance, metal/metalloid detoxification, and opportunistic survival strategies. Dominant biomarkers included *Lysobacter*, *Pseudomonas*, *Ramlibacter*, *Lacibacter*, and *Brevundimonas*. These taxa are often found in heavily impacted environments and are characterized by the ability to secrete extracellular enzymes, form protective biofilms, and resist toxicants through metal efflux systems or siderophore production [81,82]. For example, *Lysobacter* is capable of lysing other microbes through secretion of lytic enzymes and is often considered an ecological indicator of high competitive pressure [83]. Similarly, *Pseudomonas* is well known for its multidrug resistance and capacity to colonize hostile environments, often linked with pathogenesis in both plants and humans [55]. The co-dominance of these genera at L2 highlights the selective pressure exerted by long-term sulfur residue exposure and suggests a microbial community skewed toward survival, defense, and competition. At the intermediate downstream site (L3), a distinct set of biomarkers emerged, including *Nitrospira*, *Candidatus Nitrosocosmicus*, *Methyloceanibacter*, and *Rhodospirillum*. These taxa are key players in nitrogen and carbon cycling under oligotrophic or chemically constrained conditions [84,85]. The presence of both ammonia-oxidizing archaea and nitrite-oxidizing bacteria points to an active nitrifying consortium, potentially sustained by adaptation to altered redox gradients caused by smelting pollution. This pattern is consistent with reports that nitrogen-transforming communities can exhibit high resistance and functional redundancy in disturbed soils [86]. Moreover, the detection of *Candidatus Nitrososphaera* and *Rhodospirillum*, which are often found in suboxic and contaminated sediments, further supports the hypothesis that L3 represents a transitional zone, where microbial assembly is shaped by a balance between environmental stress and functional adaptation [87].

In addition to differential abundance analysis, we examined the core microbiome of sulfur-smelting soils, defined here as genera present in at least 90% of all samples and among the top 5% most abundant taxa. This analysis revealed a broad and stable community backbone composed of 710 genera (Table S1). Representative members of the core included Pseudomonadota (*Pseudomonas*, *Rhizobacter*, *Rhodospirillum*, *Lysobacter*), Actinomycetota (*Streptomyces*, *Nocardioides*, *Micromonospora*, *Actinomadura*), Acidobacteriota (*Luteitalea*, *Gemmatirosa*), Chloroflexota (*Ktedonobacter*), Bacteroidota (*Flavisolibacter*, *Chryseolinea*), Planctomycetota (*Pseudolabrys*), and Verrucomicrobiota (*Opitutus*). The persistence of these taxa across sites and depths indicates that they represent key ecological players in smelting-impacted soils. Core members such as *Pseudomonas* and *Rhodanobacter* are known for metal tolerance and biotransformation [55]; *Streptomyces*, *Micromonospora*, and related Actinomycetota contribute to organic matter turnover and intrinsic antibiotic resistance [88]. Acid-tolerant taxa from Acidobacteriota and sulfur-oxidizing groups such as *Acidithiobacillus* are likely central to maintaining ecosystem processes under acidic and metal-rich conditions [89]. These suggest that the core microbiome provides a resilient functional backbone that sustains key biogeochemical processes and supports microbial adaptation under long-term contamination stress.

To further explore the influence of soil physicochemical variables on microbial community structure, we performed redundancy analysis (RDA) (Figure S11, Tables S2 and S3). The ordination revealed clear separation of microbial assemblages along contamination gradients. Among the tested variables, As, Cr, Cd, and Ni were significantly correlated with community variation (*p* < 0.05), with Cr (r^2^ = 0.355, *p* = 0.012) and As (r^2^ = 0.328, *p* = 0.017) explaining the greatest proportion of variance. Cd (r^2^ = 0.275, *p* = 0.033) and Ni (r^2^ = 0.285, *p* = 0.029) were also significant contributors, while Cu showed weaker and non-significant effects. These findings indicate that metal/metalloid contamination, particularly As, Cr, and Cd, acts as a major environmental filter driving microbial community assembly in smelting-impacted soils. The strong explanatory power of these elements aligns with our correlation analyses of resistance and virulence gene subtypes, suggesting that the same selective pressures not only influence taxonomic composition but also shape the functional gene repertoire.

One limitation of this study is that we analyzed total concentrations of metals and metalloids, which do not directly represent the bioavailable fractions. The environmental risk and biological impact of these elements depend strongly on the forms in which they occur [90]. Metals tightly bound in stable sulfide minerals may remain relatively immobile, while those associated with carbonates, organic matter, or Fe/Mn oxyhydroxides can be more easily released under changing pH and redox conditions [91]. The soluble and weakly bound fractions exert direct selective pressure on microbial communities. Therefore, total concentrations may overestimate the actual exposure that microorganisms experience. Nevertheless, our results still provide valuable insights into overall contamination gradients and their correlations with microbial risk genes. Future work using sequential extraction or speciation analyses would be needed to better resolve the bioavailable pools and their role in shaping microbial adaptation. In addition, sulfate concentration is widely recognized as an indicator of sulfide oxidation. Sulfide minerals such as pyrite undergo microbial and abiotic oxidation, producing sulfate and sulfuric acid. These reactions drive acidification of the microenvironment and enhance the solubility and leaching of associated metals [12]. The resulting acidic leachates not only accelerate contaminant dispersion but also impose strong selective pressures on microbial communities, favoring acidophilic and metal-tolerant taxa. Although we assessed contamination primarily through total metal concentrations, incorporating sulfate measurements in future studies would provide valuable insights into the degree of sulfide oxidation and its coupling with microbial processes. Furthermore, in this study, vertical sampling was conducted at a single representative location within the smelting deposit zone, while horizontal transects were aligned along the river to capture downstream contamination gradients. This design was effective for characterizing vertical stratification and leachate-driven dispersion, but it may not fully capture spatial heterogeneity across the broader landfill area. In particular, additional vertical profiles from different deposit points and sampling on the lateral sides of the landfill would help to assess potential windborne dispersal or cross-slope migration of contaminants and associated microbial responses. Future work incorporating a more spatially extensive sampling design, with a greater number of measurement and control points, will be important for providing a more representative picture of contamination patterns and microbial community dynamics across the entire site.

## 4. Conclusions

This study investigated the spatial distribution of metal/metalloid contamination, microbial risk genes, and soil microbiomes at a long-term sulfur smelting site. Heavy metals/metalloids such as As, Cr, Cu, and Pb exhibited a pronounced enrichment trend with increasing depth. Horizontally, metal/metalloid concentrations peaked at the smelting deposit zone, while both upstream and downstream sites showed comparatively lower levels, revealing persistent vertical and horizontal pollution gradients. These contamination patterns corresponded with elevated abundances of resistance genes (ARGs, BRGs, and MRGs) and virulence factor genes (VFGs), suggesting potential co-selection driven by prolonged metal/metalloid stress. Moreover, significant shifts in microbial community composition and diversity were observed, with stress-tolerant and functionally adaptive taxa enriched in deeper and more contaminated layers. The enrichment of stress-tolerant and potentially pathogenic genera such as Pseudomonas, Acinetobacter, and Streptomyces, together with the co-occurrence of multidrug resistance and virulence gene subtypes, may serve as practical bioindicators for environmental monitoring of smelting-contaminated soils. Overall, the study offers a valuable foundation for assessing ecosystem stability and guiding bioremediation strategies in legacy smelting-impacted environments. Future research should focus on integrating bioavailability assays and mineralogical fractionation to better link metal speciation with microbial responses, as well as longitudinal and multi-omics approaches to track the dynamics of resistance and virulence genes over time. Such efforts will provide a more comprehensive understanding of how legacy contamination shapes ecological function and potential health risks.

## Figures and Tables

**Table 1:** Heavy metal/metalloid contents of soil samples.

Group	As (mg/kg)	Cr (mg/kg)	Cd (mg/kg)	Cu (mg/kg)	Ni (mg/kg)	Pb (mg/kg)
D1	12.85 ± 0.44 c	107.42 ± 4.83 bc	0.34 ± 0.01 c	158.67 ± 14.05 a	22.96 ± 0.59 c	47.64 ± 3.51 a
D2	18.96 ± 1.44 c	105.34 ± 11.55 c	0.38 ± 0.02 c	149.46 ± 7.29 ab	16.76 ± 0.50 d	50.49 ± 4.61 a
D3	31.55 ± 3.28 b	133.42 ± 14.06 ab	0.54 ± 0.03 b	131.09 ± 4.60 bc	42.56 ± 1.32 b	26.14 ± 2.05 b
D4	53.96 ± 3.91 a	153.79 ± 8.84 a	1.24 ± 0.07 a	118.67 ± 12.21 c	68.09 ± 2.81 a	26.58 ± 0.66 b
L1	13.69 ± 0.52 b	63.26 ± 6.10 c	0.13 ± 0.02 c	89.67 ± 2.25 c	16.39 ± 1.64 c	23.44 ± 0.79 c
L2	28.90 ± 2.58 a	112.03 ± 11.68 a	0.32 ± 0.01 bc	169.31 ± 11.38 a	59.75 ± 3.08 b	53.76 ± 5.19 a
L3	14.19 ± 1.21 b	84.62 ± 7.07 bc	2.36 ± 0.17 a	138.07 ± 9.46 b	57.92 ± 1.62 b	41.22 ± 4.5 b
L4	15.89 ± 1.28 b	91.84 ± 6.70 ab	0.41 ± 0.03 b	126.43 ± 13.56 b	87.87 ± 7.93 a	28.15 ± 1.05 c

Note: Different letters indicate significant differences at the *p* < 0.05 level across soil vertical layers or horizontal transects.

**Table 2:** Physicochemical properties of soil samples.

Group	pH	OM (g/kg)	TN (g/kg)	TP (mg/kg)	TK (mg/kg)	AN (mg/kg)	AP (mg/kg)
D1	4.33 ± 0.51 a	23.17 ± 2.29 a	1.03 ± 0.05 b	1365.53 ± 11.03 a	2291.76 ± 205.99 b	134.06 ± 10.35 a	114.14 ± 8.51 c
D2	4.03 ± 0.43 a	21.66 ± 1.56 a	1.07 ± 0.06 b	1486.72 ± 53.92 a	2888.83 ± 234.29 a	102.19 ± 9.34 b	118.86 ± 6.76 bc
D3	4.41 ± 0.48 a	21.55 ± 1.49 a	1.47 ± 0.12 a	1573.79 ± 80.44 a	1858.57 ± 156.6 b	108.53 ± 11.75 ab	136.58 ± 12.92 ab
D4	5.02 ± 0.21 a	23.28 ± 2.36 a	1.12 ± 0.05 b	1418.03 ± 85.47 a	1941.7 ± 120.46 b	85.04 ± 8.86 b	157.67 ± 7.27 a
L1	4.88 ± 0.49 a	24.9 ± 2.57 a	1.44 ± 0.1 b	1236.15 ± 44.21 b	4960.25 ± 446.28 b	146.98 ± 7.74 b	141.43 ± 14.88 b
L2	4.07 ± 0.24 b	24.61 ± 2.27 a	0.48 ± 0.05 d	1540.53 ± 70.04 a	2387.23 ± 185.92 c	38.78 ± 2.78 d	181.21 ± 4.75 a
L3	6.04 ± 0.38 a	23.59 ± 2.21 a	2.36 ± 0.08 a	1537.33 ± 142.28 a	7470.56 ± 664.58 a	235.8 ± 23.73 a	205.03 ± 18.84 a
L4	6.46 ± 0.38 a	24.7 ± 1.01 a	0.99 ± 0.04 c	1136.46 ± 89.9 b	3916.87 ± 330.51 b	96.77 ± 6.46 c	112.98 ± 7.4 b

Note: Different letters indicate significant differences at the *p* < 0.05 level across soil vertical layers or horizontal transects.

## Data Availability

The original contributions presented in this study are included in the article/Supplementary Materials. Further inquiries can be directed to the corresponding authors.

## Supplementary Materials

The following supporting information can be downloaded at: https://www.mdpi.com/article/10.3390/microorganisms13092010/s1, Figure S1. Sampling sites. A map showing the location and landscape of soil sampling sites. Figure S2. Abundance of the main soil ARG subtypes along the vertical layers. Figure S3. Abundance of the main soil ARG subtypes along the horizontal transects. Figure S4. Abundance of the main soil BRG subtypes along the vertical layers. Figure S5. Abundance of the main soil BRG subtypes along the horizontal transects. Figure S6. Abundance of the main soil MRG subtypes along the vertical layers. Figure S7. Abundance of the main soil MRG subtypes along the horizontal transects. Figure S8. Abundance of the main soil VFG subtypes along the vertical layers. Figure S9. Abundance of the main soil VFG subtypes along the horizontal transects. Figure S10. Spearman correlation between heavy metals/metalloids and subtypes of resistance and virulence genes. Significance levels are indicated as * *p* < 0.05, ** *p* < 0.01, and *** *p* < 0.001. Figure S11. Redundancy analysis (RDA) illustrating the relationships among heavy metals/metalloids, soil physicochemical properties, and microbial community composition. Table S1. Core microbiome of sulfur-smelting soils. Table S2. Redundancy analysis (RDA) scores of metals and physicochemical variables. Table S3. Redundancy analysis (RDA) scores of microbial community.
