# Supplementary material for: Spatial Differentiation of Heavy Metals/Metalloids, Microbial Risk Genes and Soil Microbiota in a Sulfur-Contaminated Landscape
Source: Microorganisms. 2025 Aug 28;13(9):2010. doi: 10.3390/microorganisms13092010 (PMC12473080; doi:10.3390/microorganisms13092010)
Supplement: Supplementary file 1 [file microorganisms-13-02010-s001.zip › microorganisms-3753766 Supplementary Figures.pdf]

Supplementary materials for

Spatial differentiation of heavy metal, microbial risk genes and soil microbiota in a sulfur-contaminated landscape

Lina Li [1,2], Jiayin Zhao [3], Chang Liu [2], Yiyan Deng [2], Yunpeng Du [2], Yu Liu [3], Yuncheng Wu [3], Wenwei Wu [2,*] and Xuejun Pan [1,*]

[1] Faculty of Environmental Science and Engineering, Kunming University of Science and Technology, Kunming 650500, China

[2] Yunnan Academy of Ecological and Environmental Sciences, Kunming 650034, China

[3] Nanjing Institute of Environmental Sciences, Ministry of Ecology and Environment, Nanjing 210042, China

[*] Corresponding authors at: Faculty of Environmental Science & Engineering, Kunming University of Science and Technology, Kunming 650500, China.

E-mail address: wuwwp@163.com (W.W.); xjpan@kust.edu.cn (X.P.)

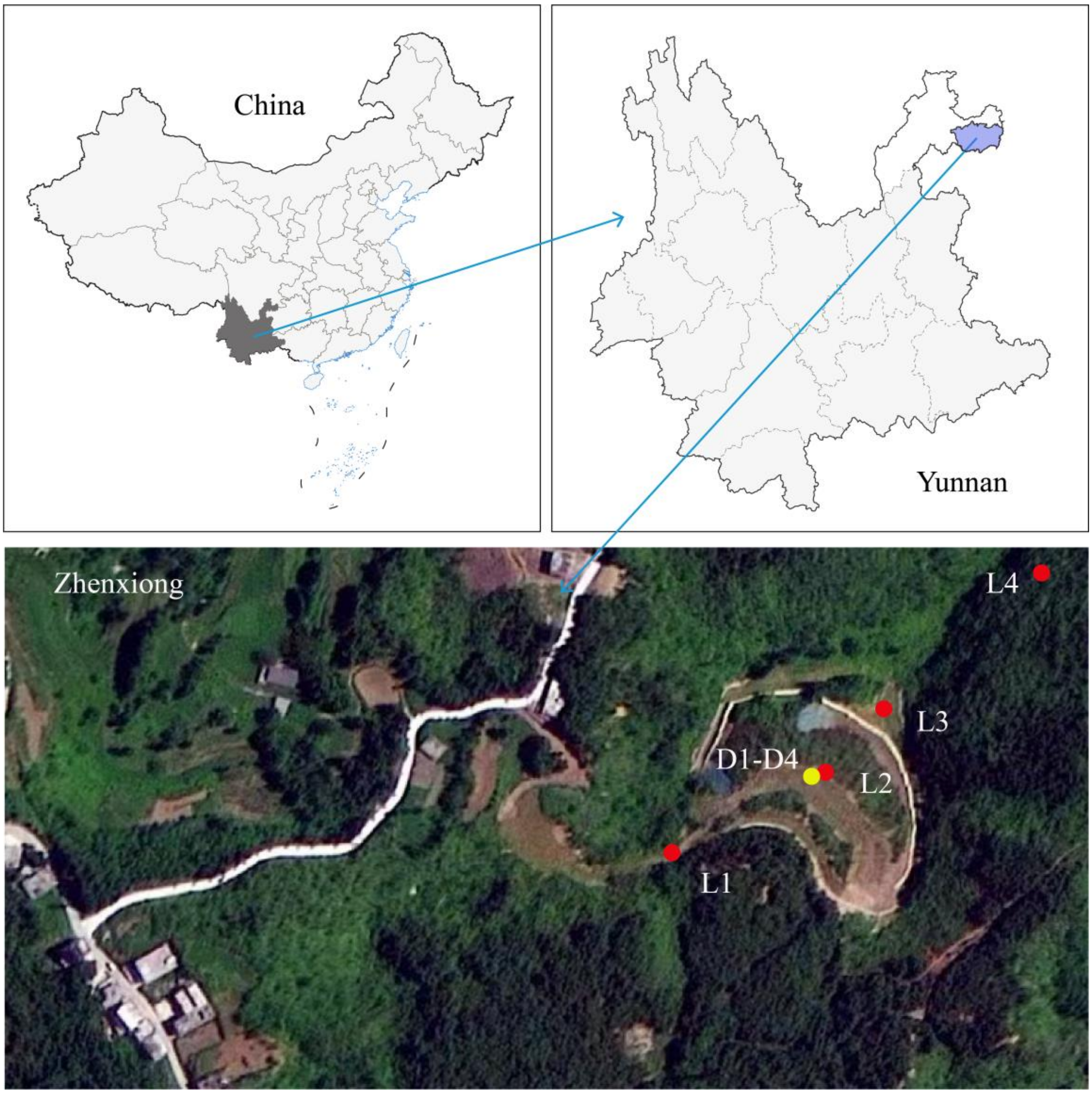

Figure S1 Sampling sites. A map showing the location and landscape of soil sampling sites.

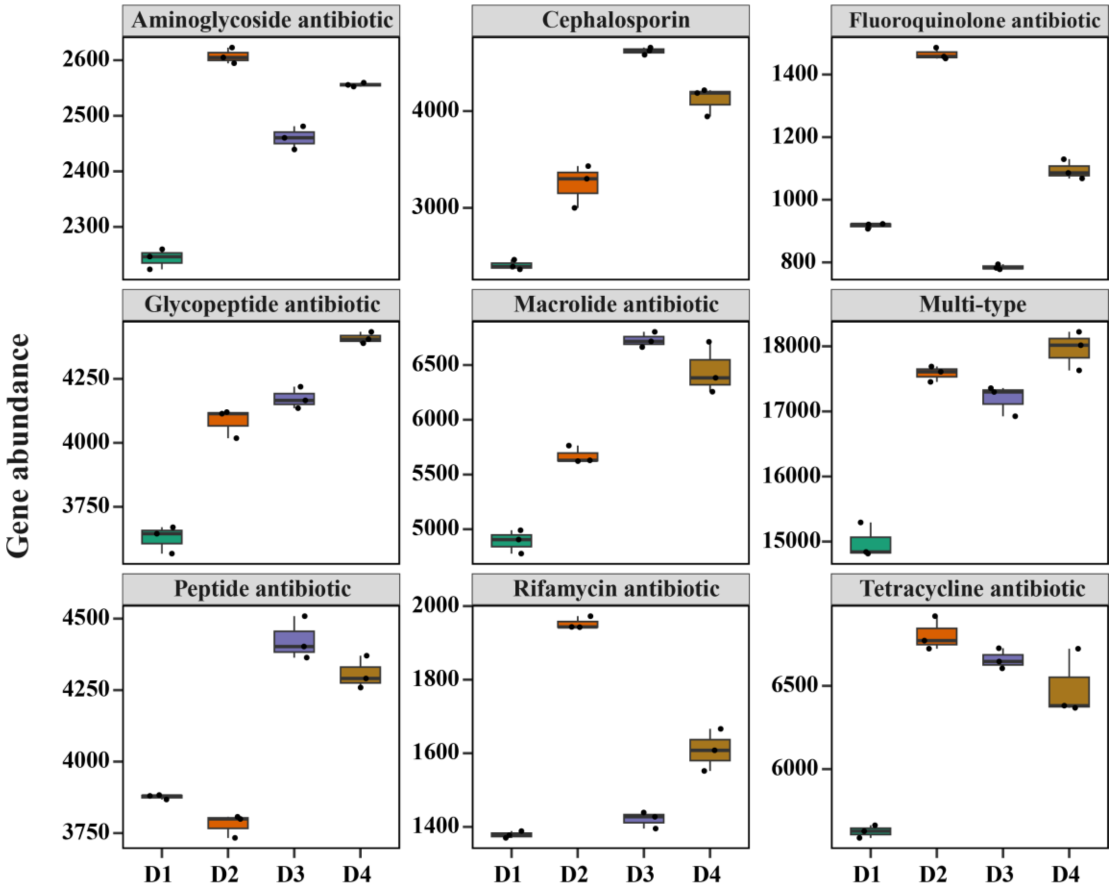

Figure S2. Abundance of the main soil ARG subtypes along the vertical layers.

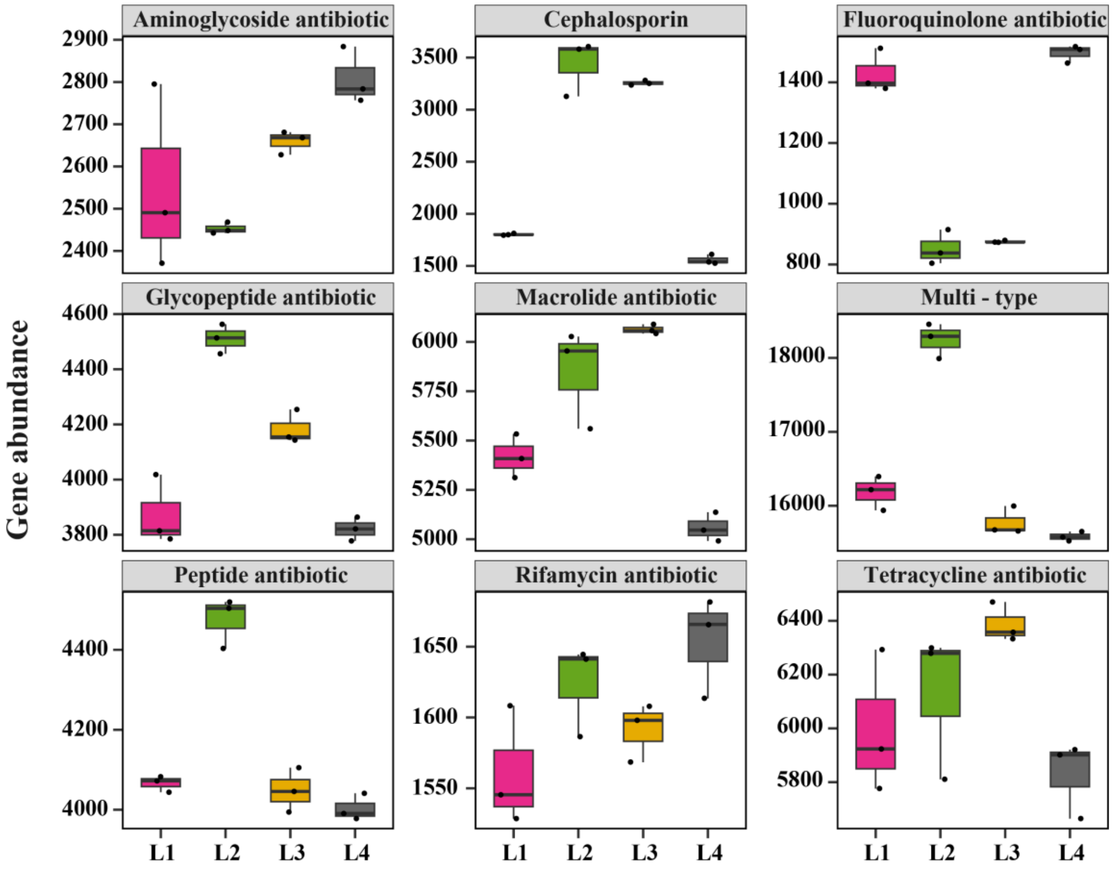

Figure S3. Abundance of the main soil ARG subtypes along the horizontal transects.

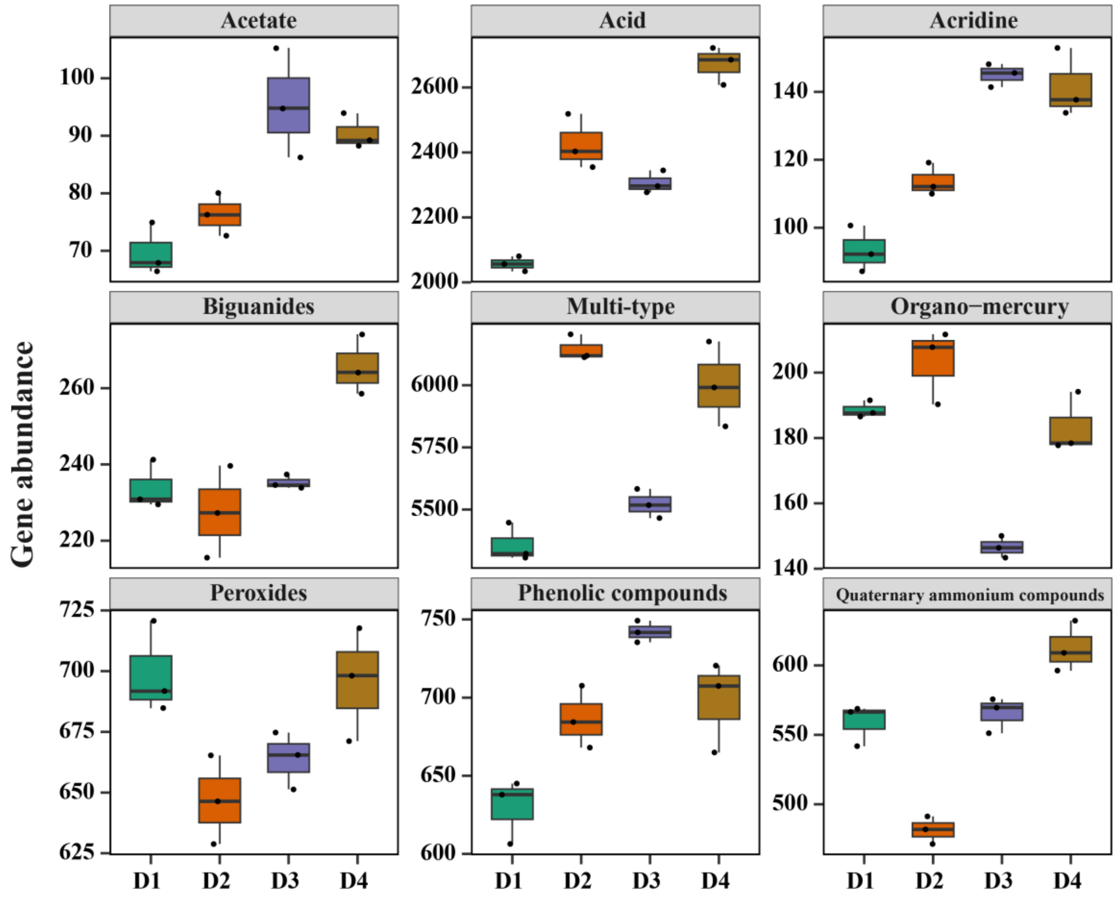

Figure S4. Abundance of the main soil BRG subtypes along the vertical layers.

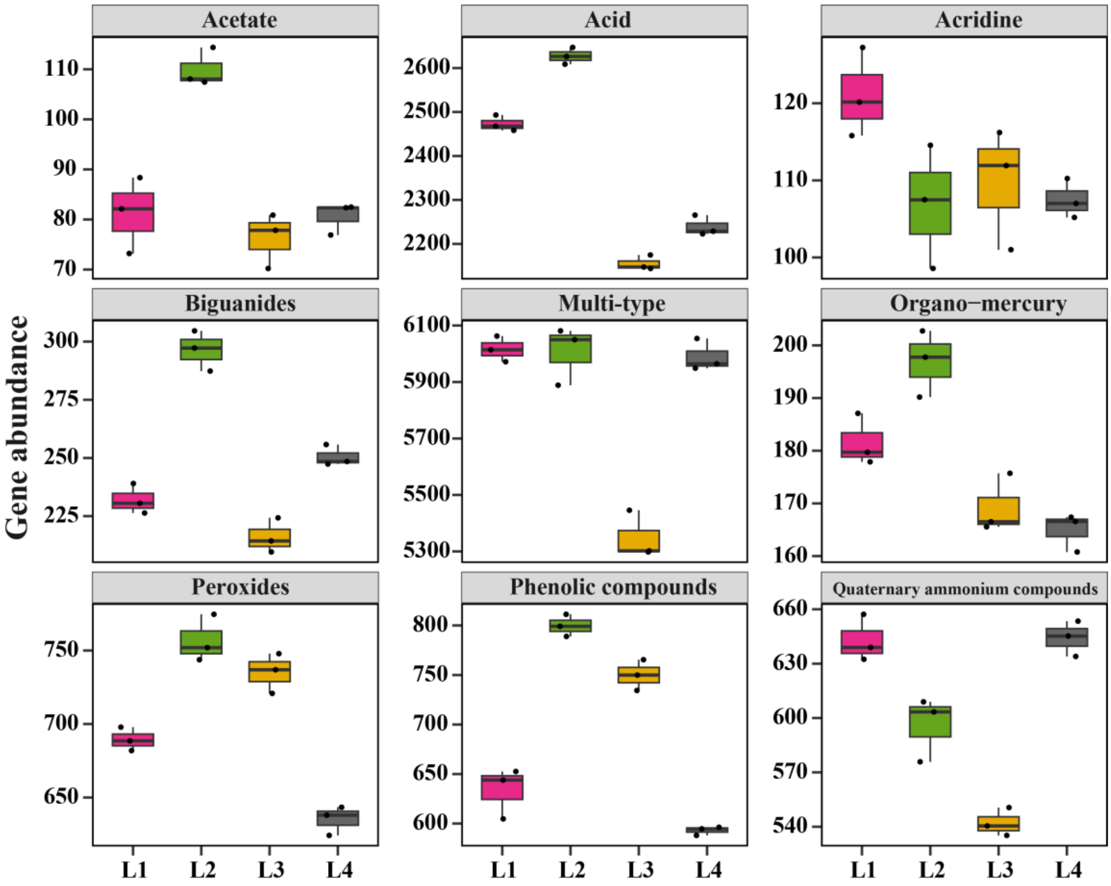

Figure S5. Abundance of the main soil BRG subtypes along the horizontal transects.

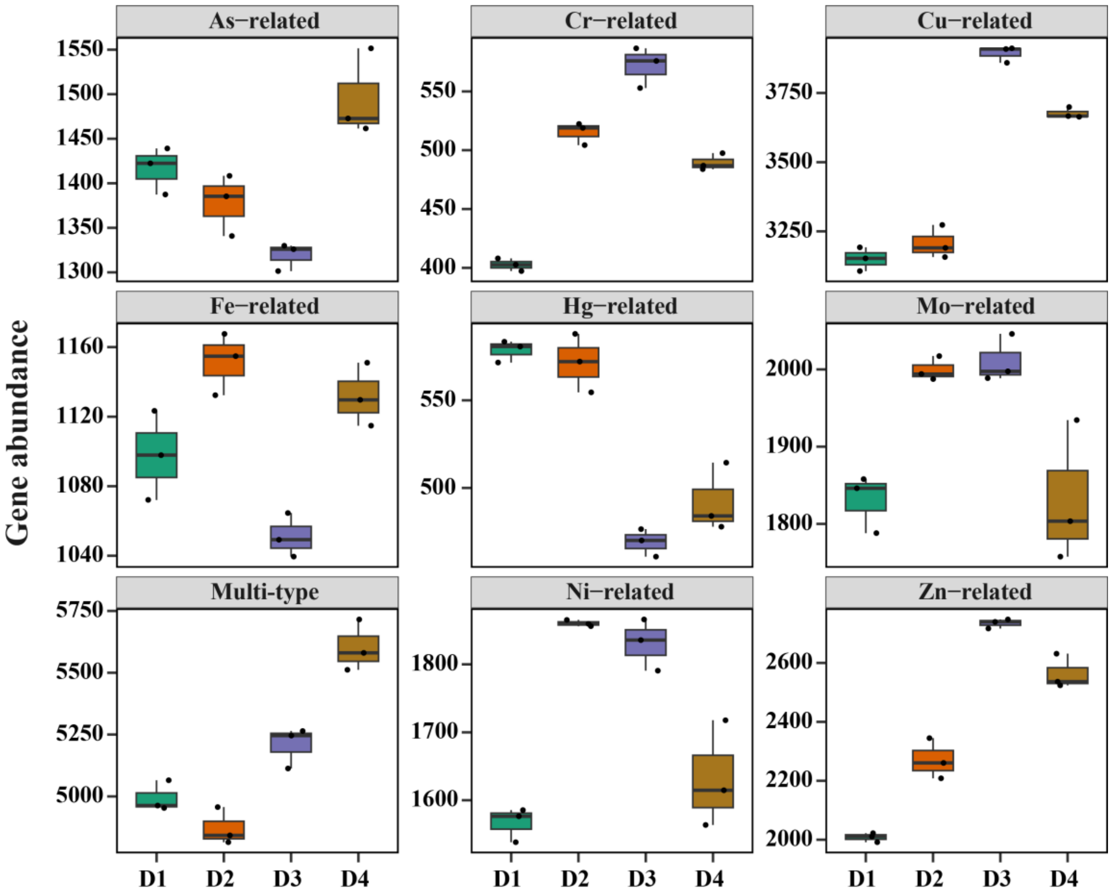

Figure S6. Abundance of the main soil MRG subtypes along the vertical layers.

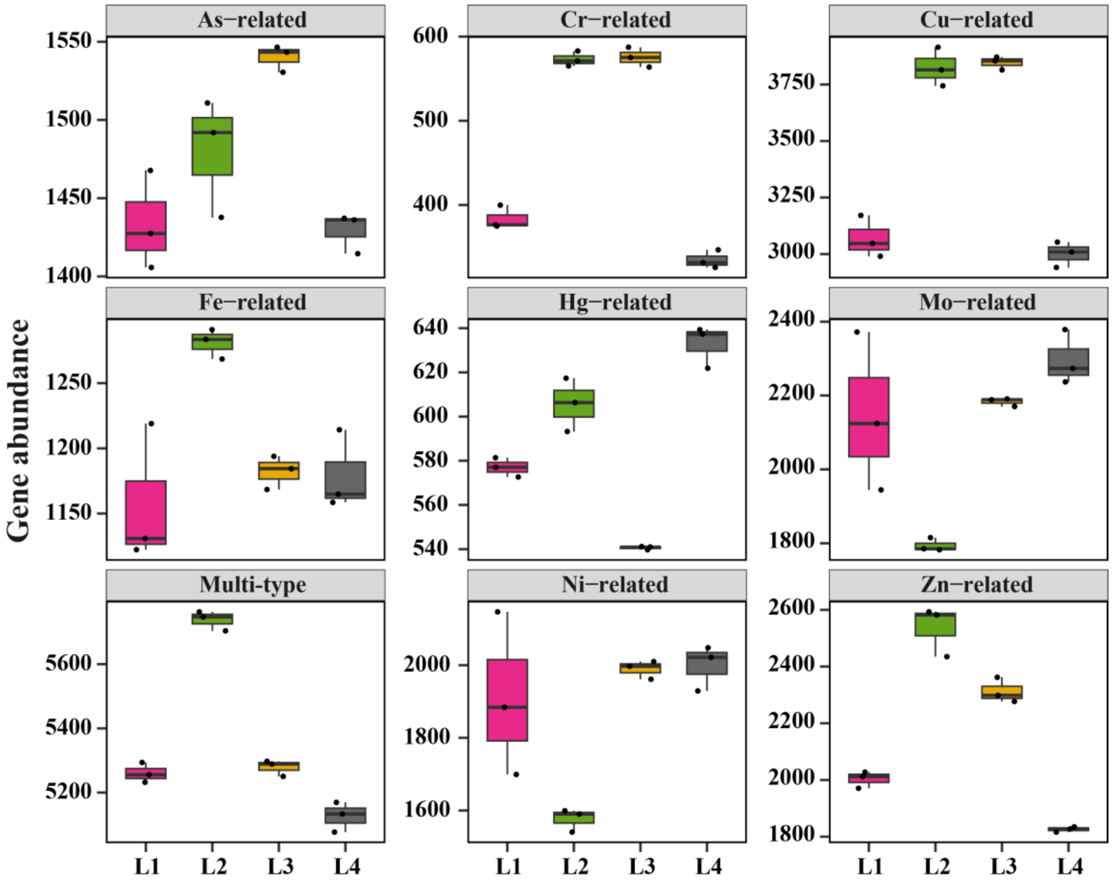

Figure S7. Abundance of the main soil MRG subtypes along the horizontal transects.

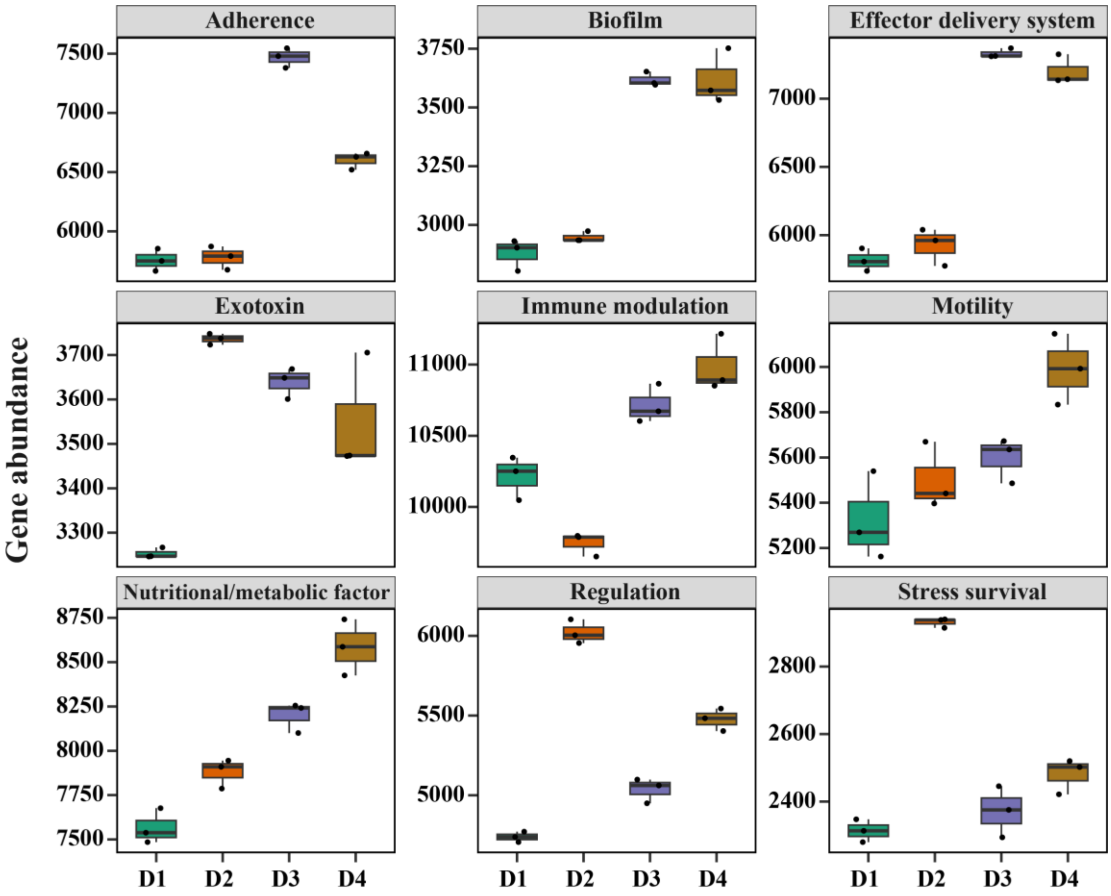

Figure S8. Abundance of the main soil VFG subtypes along the vertical layers.

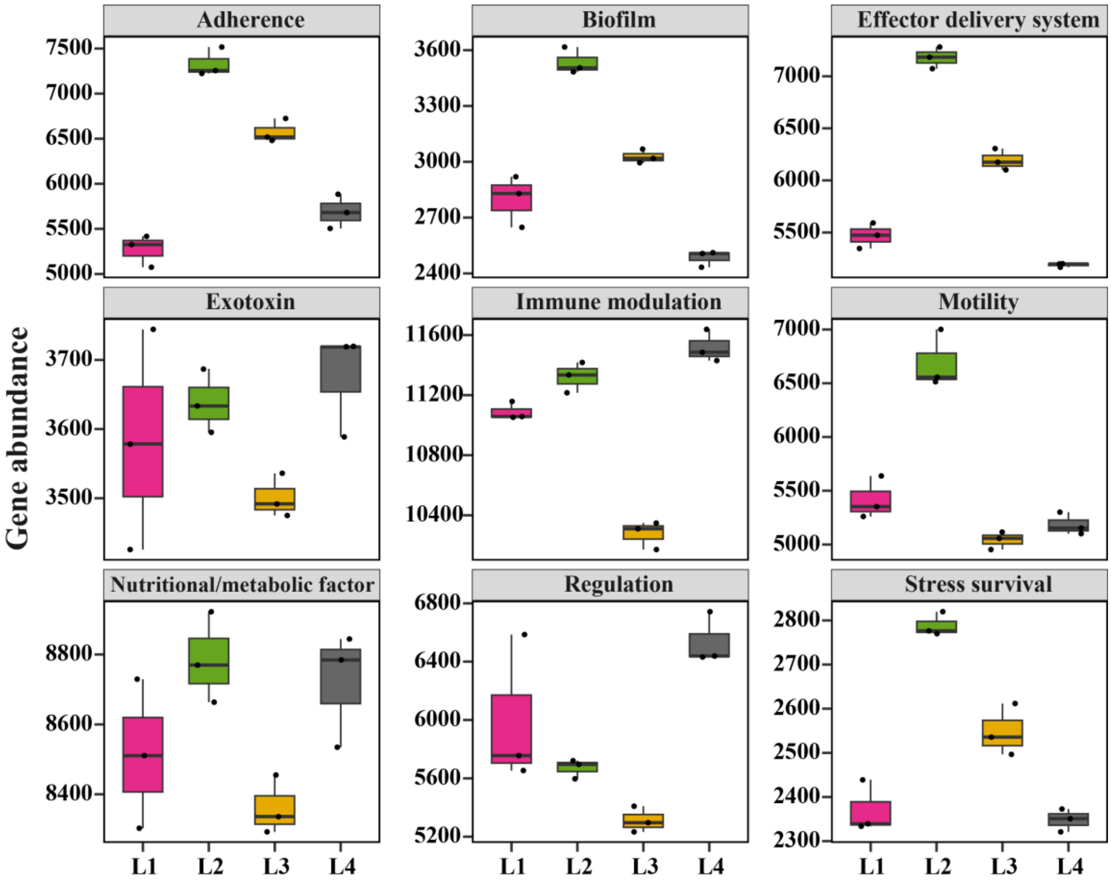

Figure S9. Abundance of the main soil VFG subtypes along the horizontal transects.

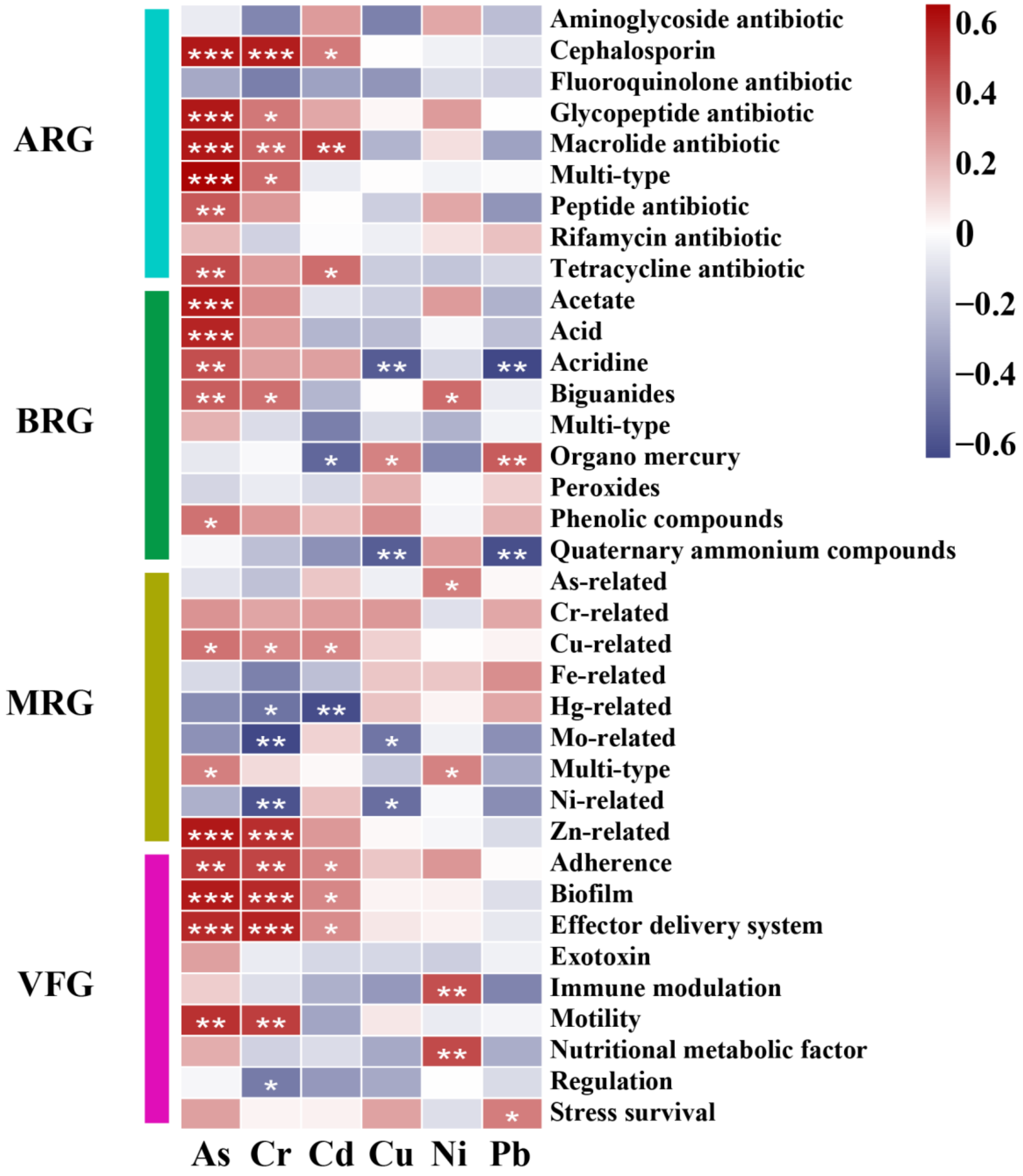

Figure S10. Spearman correlation between heavy metals/metalloids and subtypes of resistance and virulence genes. Significance levels are indicated as *p < 0.05, **p < 0.01, and *p < 0.001.

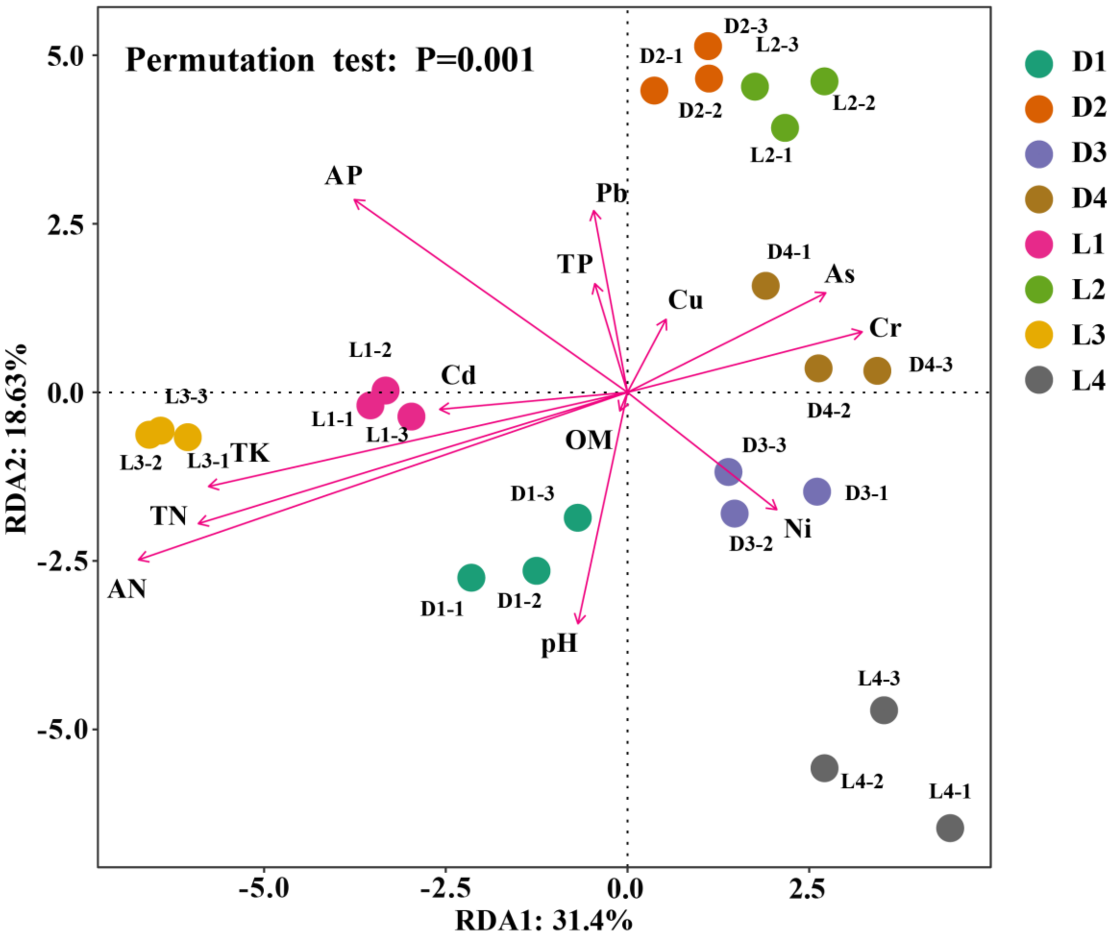

Figure S11. Redundancy analysis (RDA) illustrating the relationships among heavy metals/metalloids, soil physicochemical properties, and microbial community composition.
